# Rationale and design of the research project of the South Florida Center for the Reduction of Cancer Health Disparities (SUCCESS): study protocol for a randomized controlled trial

**DOI:** 10.1186/1745-6215-15-299

**Published:** 2014-07-23

**Authors:** Olveen Carrasquillo, Sheila McCann, Antony Amofah, Larry Pierre, Brendaly Rodriguez, Yisel Alonzo, Kumar Ilangovan, Martha Gonzalez, Dinah Trevil, Margaret M Byrne, Tulay Koru-Sengul, Erin Kobetz

**Affiliations:** Departments of Medicine, University of Miami Miller School of Medicine, Locator code C223, 1120 NW 14th Street, Miami, FL 33136 USA; Department of Public Health Sciences, University of Miami Miller School of Medicine, 1120 NW 14th Street, 10th floor, Miami, FL 33136 USA; Sylvester Comprehensive Cancer Center, University of Miami, Miller School of Medicine, Clinical Research Building, 3rd floor, 1120 NW 14th Street, Miami, FL 33136 USA; Health Choice Network, 9064 N.W. 13 Terrace, Miami, FL 33172 USA; Center for Haitian Studies, 8260 NE 2nd Ave, Miami, FL 33138 USA

**Keywords:** Cervical cancer, Community-based participatory research, Haitian, Health disparities, Hispanic, Human papilloma virus, Immigrant, Minority, Screening

## Abstract

**Background:**

In the United States certain minority groups, such as racial/ethnic immigrant women, are less likely than non-Hispanic White women to be screened for cervical cancer. Barriers to such care include health insurance, cost, knowledge, attitudes, health literacy, and cultural norms and practices. Among the most promising approaches to increase screening in these groups are patient navigators that can link women to sources of appropriate care. Another recent promising approach is using human papilloma virus (HPV) self-sampling. In this manuscript, we describe our National Cancer Institute-sponsored study testing such approaches among immigrant minority women.

**Design:**

The South Florida Center for the Reduction of Cancer Health Disparities (SUCCESS) is conducting a three-arm randomized trial among Hispanic, Haitian, and African American women in Miami-Dade County. Community health workers (CHW) based in each of three communities are recruiting 200 women at each site (600 total). Eligibility criteria include women aged 30–65 years who have not had a Pap smear test in the last 3 years. Prior to randomization, all women undergo a standardized structured interview. Women randomized to public health outreach, Group 1, receive culturally tailored educational materials. Women in Group 2 receive an individualized comprehensive cervical cancer CHW-led education session followed by patient navigation to obtain the Pap smear test at community-based facilities. Women in Group 3 have the option of navigation to a Pap smear test or performing HPV self-sampling. The primary outcome is self-report of completed screening through a Pap smear test or HPV self-sampling within 6 months after enrollment.

**Discussion:**

SUCCESS is one of the first trials testing HPV self-sampling as a screening strategy among underserved minority women. If successful, HPV self-sampling may be an important option in community outreach programs aimed at reducing disparities in cervical cancer.

**Trial registration:**

Clinical Trials.gov # NCT02121548, registered April 21, 2014.

## Background and rationale

Cervical cancer disproportionately affects women of color. In 2010, the cervical cancer incidence rate per 100,000 was 9.8 for Black women and 9.6 for Hispanic women versus 7.2 for White women [1]. The cervical cancer mortality per 100,000 women was 3.9 for Blacks and 2.6 for Hispanics versus for 2.1 non-Hispanic women [1]. In Florida, 67% of Black women presented with cervical cancer at an advanced stage versus 49% of White women [2]. Cervical cancer is also a highly preventable cancer. Cytological screening (Papanicolaou smear or Pap smear test) among women aged 21 to 65 years every 3 years or for women aged 30 to 65 years substantially reduces cervical cancer incidence and mortality [3]. With such enhanced public health promotion efforts, the vast majority of women in the United States (US) now have cervical cancer screening at least every 3 years [4]. As a result, since 2000, cervical cancer mortality and incidence have been decreasing and racial and ethnic disparities have narrowed [1].

Despite these gains, approximately 50% of cases of cervical cancer are diagnosed in women who have not been screened for more than 5 years. Further, not all women have benefited equally from these gains and certain groups remain vulnerable. Most at risk are immigrant women, including Latinas, Caribbean Blacks, and certain Asian subgroups. As an example, nationally among Latina immigrants, 70% had not completed a Pap smear test in the last 3 years [5]. Haitian women are particularly vulnerable with only 44% of such women having a Pap smear test in the last three 3 years [6]. These rates are substantially lower than the national cervical cancer screening rates of 87% [4]. Important risk factors for not being screened are immigration status, lack of insurance, length of time in US, language, and low levels of acculturation [4–6]. Additionally, among some minority and immigrant groups, socio-cultural perceptions of health, preferences for non-traditional health modalities, and historical distrust of outsiders also plays a role in screening disparities [6].

To date, one of the most promising approaches to increase cancer screening in this population has been using community health workers (CHWs). CHWs, also known as lay health workers, are community members without formal health care education who serve as a link between patients and providers to promote health among groups traditionally lacking adequate health care. A few well designed studies have found that CHWs are effective at increasing screening rates [7, 8]. However, the absolute increase is modest with an average median increase of 10 percentage points over baseline screening rates in the target communities [9].

Thus, there is a need for additional novel strategies to increase screening in these groups. One promising approach builds on the role of detection for the presence of carcinogenic strains of the human papilloma virus (HPV) as part of cervical cancer screening [10, 11]. Nearly all cases of cervical cancer are the result of infection with oncogenic strains of the HPV. Until a few years ago, HPV detection was primarily used for reflex testing of women whose Pap smear test results were indeterminate (atypical cells of unclear significance, i.e., ASCUS). Subsequently, in 2012, the US Preventive Services Task Force (USPSTF) recommended concomitant use of HPV screening with Pap smear cytology for cervical cancer screening among all women. Then, in 2014, an FDA advisory committee unanimously recommended that the HPV test using polymerase chain reaction (PCR) could be used as the initial test for cervical cancer screening [12].

One particular characteristic of HPV testing that makes it important as a tool to reach underserved women is that women can self-collect their own swabs without having to undergo a traditional pelvic exam. A recent meta-analysis found that the sensitivity and specificity of self-collected HPV samples were similar to clinician-collected samples when samples were analyzed using PCR-based testing and only slightly lower when using signal amplification-based assays [13]. Another study among Mexican women showed that self-testing was as effective as cytology screening even when scaled up in large populations [14]. Thus, increasingly, HPV self-sampling is being recommended as an important strategy to reach women not currently being screened. In the US, studies have also shown the feasibility of this approach as an outreach strategy for underserved women [15, 16]. However, whether this approach is superior to standard outreach methods, such as public health education campaigns or approaches using CHWs, is unclear.

### Objectives

To address this gap in knowledge, our study objective is to conduct a randomized trial that seeks to examine the effectiveness of a CHW-led outreach strategy that includes HPV self-sampling at increasing Pap smear screening among minority women who are not up to date on their Pap smear testing. This approach is being compared to standard public health outreach using culturally-tailored health education brochures and to a CHW-led outreach intervention that includes individualized patient navigation to health care facilities providing low cost screenings.

## Methods

### Participants, interventions, and outcomes

#### Overview and trial design

Our study is a randomized controlled trial of 600 women in three communities in Miami-Dade County (South-Dade, Little Haiti, and Hialeah). Minority women, aged 30 to 65, who have not completed a cervical cancer screening in the past 3 years and who agree to participate in the study have a 30-minute intake visit by one of our two bilingual research assistants (RA). Once they complete our research intake survey, they are randomized into one of three possible interventions. The public health outreach group receives culturally tailored cervical cancer health education materials and brochures indicating locations where low cost Pap smear screening is available (Group 1). The second group undergoes a comprehensive CHW outreach intervention that includes home visits and patient navigation to help women obtain low cost Pap smear screening in their community. The third group receives the comprehensive CHW intervention and the opportunity to have home HPV self-sampling at the time of visit. The primary outcome is self-reported cervical cancer screening (either Pap smear test or via self-sampled HPV screening) determined at a 6-month follow-up assessment done by a RA blinded to study allocation.

#### Conceptual approach

Conceptually, the design of our intervention is theoretically grounded on contemporary models specifically designed to facilitate the design and understanding of successful interventions to reduce disparities in health care among diverse communities [17]. The critical focus of such approaches is that persons reside within a community and not as patients in a healthcare system. Thus, critical for success of an intervention is linking persons in underserved settings with effective health care delivery. Equally important in our approach is the use of principles of community-based participatory research. This strategy has emerged as one of the most promising frameworks towards reducing health disparities [18]. In SUCCESS, our two key community partners are: 1) Health Choice Network of Florida (HCN), which includes two Federally Qualified Health Center (FQHCs) systems, and 2) the Center for Haitian Studies. In the study, these community partners are full participants in all phases of the project including the conception, design, conduct, interpretation of findings, and dissemination of results. Through several face-to-face meetings and conference calls, the community partners had substantial input in the study design including: 1) selection of geographic sites, 2) design of the CHW interventions (recruitment approach, conduct of home visits), 3) evaluative metrics (inclusion of measures of satisfaction and access to care), 4) ethical issues (all participants are offered HPV self-sampling at the end of their study participation), and 5) dissemination strategies (presentations to their boards, lay summaries in their newsletters). In addition, the intervention is being delivered by the CHWs, who are hired and based in these organizations rather than at the academic health center. Nearly half the research budget is allocated to these partners.

#### Study setting

For the study, the target communities we chose reflect the ethnic diversity in Miami-Dade. One is Little Haiti; Florida is home to more than a third of all Haitians in the US and Miami has the highest concentration of Haitians in the state. Little Haiti, in the northern section of Miami, has an estimated 200,000 Haitians. Most are recent immigrants who are low income, have limited formal education, and restricted access to the formal healthcare system. In addition, given their double minority status (Black and Kreyol speaking), the integration of Haitians is often slower and more challenging than that of other immigrant groups. In Little Haiti, we are working with the Center for Haitian Studies, one of the community’s leading social service organizations, which also operates a community-based healthcare facility. Our second community is Hialeah which is a separate city within Miami-Dade County. This city includes over 200,000 residents of which 94% self-identify as Hispanic and 62% of which are Cuban. As suggested by HCN, in Hialeah, we are working with Citrus Health Network Inc., the largest FQHC in Hialeah. The third target area is the southern portion of Miami-Dade county (South-Dade) which is much less urban than the above two communities. This is a very mixed landscape, including areas closer to the city composed of large low-rise public housing complexes, factory and industrial warehouses, and further out more rural agricultural areas with a large proportion of migrant farm workers. The area is also very ethnically diverse including large multi-ethnic enclaves of Latinos and Blacks, including some Haitians. In this community, we are working with Community Health of South Florida Inc. This is a FQHC network that includes several facilities and serves as the major provider of healthcare services to medically indigent residents in southern Miami-Dade County.

In each community, we are working with community advisory boards (CABs) composed of residents who represent organizations focused on health issues and socio-economic services. The CABs also include individuals who are cancer survivors and relatives or friends of persons who have been stricken with cancer. The CABs meet approximately every two months and these groups have been influential in identifying strategies for recruitment of study participants.

### Eligibility criteria

#### Inclusion criteria

To be eligible, women must be living in one of the three target communities and self-identify as Haitian, Hispanic, and/or Black. They must be aged 30 to 65 years old and not have had had a Pap smear test in the last 3 years. Women under 30 years of age have a high false positive HPV rate due to transient HPV infections. Thus, the HPV test is not recommended for women under 30 years old.

#### Exclusion criteria

A woman is not eligible to participate in the study if she i) reports having had a hysterectomy, ii) has a history of cervical cancer, iii) plans to move out of the neighborhood during the next 6 months, or iv) is currently enrolled in any other cancer prevention/outreach related study.

### Intervention

#### The CHWs

In all three arms of our study, participants are initially identified for participation and receive the intervention with the assistance of our three study CHWs. All three CHWs in the project had some experience as outreach workers but did not have any formalized health care degrees or training. All of the CHWs reflect the ethnic and racial representation of their assigned community: two are Hispanic and one is Haitian. They completed two weeks of training using modules that covered core CHW competencies [19, 20], cancer education and outreach [21], cervical cancer and HPV, and cancer clinical trials [22]. They also completed the University of Miami-required courses on human subject protections and HIPAA training using the Collaborative Institutional Training Initiative [23].

#### Intervention Group 1 – Public health community outreach

As part of our overall efforts aimed at reducing cervical cancer disparities, CHWs in each targeted community have been engaged in public health outreach. These activities include cervical cancer education campaigns over local ethnic radio stations and articles in local ethnic newspapers. The CHWs have also developed health education materials, such as brochures and fact sheets, which they distribute at events such as health fairs, community events, and other such gatherings. These are primarily based on existing materials from the National Cancer Institute (NCI) as well as other sources, such as the American Cancer Society. With feedback and suggestions from our CABs and health educators, these materials are tailored and adopted to the local culture, characteristics, and language of each community. The materials include information about the Pap smear test, why it is important, existing healthcare sources in each community, and how to go about making appointments for a Pap smear. In our study, women randomized to Group 1 are given copies of these brochures at the end of the initial intake visit. They are told the material has information on cervical cancer screening and they are encouraged to read the material. At 3 months these women also receive a brief phone call for continuity and retentions purposes reminding them of the study, obtaining any updated contact information and reminding them that at 6 months they will be called to schedule a follow-up visit by the RA.

#### Intervention Group 2 – CHW individualized health education and patient navigation

Each participant randomized to this arm receives a one-to-one health education session of approximately 1 hour. If randomized to this group, CHWs contact participants to schedule the session at a mutually convenient location. With input from professionally trained health educators and our clinical partners, the study group developed an evidence-based implementation guide as a model for conducting one-to-one health education sessions. The intervention strategies involve three overlapping domains: i) health education on cervical cancer screening, ii) motivation to encourage women to complete screening, and iii) navigation to access screening services. Study guidelines emphasize the importance of CHW judgment as to content and delivery to tailor the approach for each woman and her unique circumstances. The session begins with introductory comments followed by probing question to help individualize the intervention. Probing questions begin with generalized inquiries about what each woman knows about Pap smear tests to more targeted items asking about reasons for not having Pap tests. Based on the answers, the CHW decides how to target the intervention strategy. The CHW guide also suggests courses of action based on potential participant responses for each to consider as examples. For some women, the CHWs may need to spend time providing basic health education including explaining basic anatomy and concepts about cancer and screening, such as the relationship of early detection to reducing cancer-related mortality. For others, the discussion may also include an expanded discussion of the relationship of HPV to cervical cancer risk. This is facilitated with education materials the CHW adapts from our existing materials or other NCI resources, including anatomical graphics, brochures, and flipcharts.

#### Navigation

In addition to health education, a major role for the CHW is to offer women practical assistance that would facilitate her access to screening services. Navigation services may range from explanation of services offered at our participating clinical sites, availability of sliding fees and specific programs for cervical cancer screening for the uninsured (such as the Center for Disease Control early detection breast and cervical cancer screening programs), and assistance with making/scheduling the clinical appointments for the participant. On a case by case level, CHWs may provide assistance with other issues such as transportation (bus routes), potential language, cultural, or even child care issues, and, if requested, accompany the woman to screening appointments. CHWs also make appointment reminder calls the day prior. Within 2 weeks after a woman has the Pap smear screening, CHWs will contact the participant by phone to see if she requires any further assistance. If patients have not been notified of the results, with patient consent, the CHW tries to obtain the information from the FQHC and, with provider approval, may relay findings to the patients (all sites use electronic records). CHWs also play an important role by ensuring that each woman understands her test results. She may also provide further navigation by assisting with additional follow-up as needed, such as for colposcopy (available at each of our sites).

#### Intervention Group 3: HPV self-sampling option

Women randomized to this arm receive the same health education session as Group 2. In addition, the education includes information on HPV self-sampling as an alternative screening method. They receive an offer of choosing between the HPV screening at the conclusion of the educational session or navigation services to a traditional Pap smear test at a local healthcare facility. If the woman chooses the HPV self-sampling strategy, the CHW provides detailed instructions to complete the sample collection. Using standardized operating procedures and study-specific visual aids and flipcharts we developed, they teach women the proper self-sampling collection technique. Women complete the test in a bathroom while the CHWs wait outside to answer any questions that may arise during the process.

We are using the HPV self-sampling device developed by Preventive Oncology International and the National Institutes of Health, which is a nylon swab 2 cm in diameter and 15 cm in length. Based on prior feedback on comfort and ease of use from earlier study participants, we are using the sampler without the outer sheath. After the woman collects the sample using the swab, she removes the swab out of her vagina and gives it to the CHW. The CHW puts the swab in a pre-labeled liquid media vial (ThinPrep, Holigic Inc., Bedford, MA, US) stirs the sample, caps the bottle, and stores the sample in a re-sealable plastic bag in a locked cabinet. Once a week, the CHW delivers the samples to the University of Miami’s Department of Pathology for processing. The sample is then sent to an outside CLIA approved laboratory (Quest Diagnostics Inc.) for HPV testing. Initially our specimens were being processed by Quest using the Cervista HPV Invader Assay (Holigic In.) [24]. This was later changed to APTIMA HPV Assay (GenProbe Inc.). The former tests for HPV using a DNA based two-step signal amplification method and the latter tests for mRNA using a three step transcription-mediated amplification assay [25]. Both assays test for the 14 HPV strains which have been identified as high risk HPV by the International Agency for Research on Cancer [26] (i.e., 16, 18, 31, 33, 35, 39, 45, 51, 52, 56, 58, 59, 66, 68). They have similar sensitivities for detection of cervical intra-epithelial neoplasia (CIN2+) [27].

After the specimen is collected, the CHW asks the woman a few additional questions concerning her experience with the procedure, including ease of use, perceived correctness of device use, degree of pain associated with use, and willingness to recommend the device to family or friends as an alternative to Pap smear testing. The CHW also stresses the importance of seeing a health care provider to address any other health issues she identified during their conversations. The CHW also advises the woman that a positive result (i.e., positive for HPV high risk), will require follow-up up to ensure she receives recommended medical care. In rare cases where a submitted sample is deemed as quantity not sufficient by the laboratory, CHW will ask the woman to undergo a second HPV sampling.

#### Primary outcome

The primary outcome is self-report of completion of a Pap smear or HPV test since the initial evaluation. We will assume that women who were lost to follow-up (no further contact) did not complete a cervical cancer screening. However, participants successfully contacted by phone but who declined in-person follow-up evaluation are asked if they completed a Pap smear or HPV test. Data obtained from this phone call will be included in a sensitivity analysis on our primary outcome.

#### Secondary outcomes

There will be two secondary outcomes. The first is cervical cancer knowledge. Women in Groups 2 and 3 receive a 1-hour health education session. Thus, changes in cervical cancer knowledge from baseline to follow-up will be compared across the three groups. For each woman, the change in proportion of questions answered correctly will be examined.

The other secondary outcome will be access to care. Although improving access to care is not specifically part of the intervention, conceptually, a major role for CHWs is linking women in their communities to health care. Thus, as a secondary outcome we will determine the impact of our CHW intervention at improving access to healthcare. This will include an analysis of survey questions regarding having health insurance, a usual source of preventive care, and number of visits to a provider within the last year. By including this as a secondary outcome, it will help us examine whether access to care improved among participants having a CHW. This data will also help to address concerns that women in Group 3 who receive negative results from their completed HPV may believe that they are healthy and do not need to see a health care provider. That is, it will allow us to examine whether access among participants in Group 3 remained the same as those in Group 2.

#### Sample size

**Figure 1:**
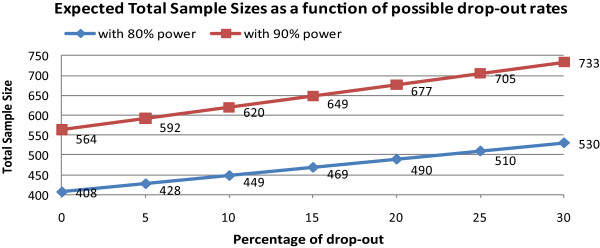
**SUCCESS sample size.** Legend: Figure shows the sample size needed (Y axis) to achieve statistical significance with 80% and 90% power, based on varying estimates of attrition (X axis).

In the study, we are recruiting 600 women from three study sites. The sample size was chosen to detect effects associated with a 20 point increase in the proportion of women screened in Group 2 (CHW) versus Group 1 (outreach) and an additional 15 percentage point increase in Group 3 (HPV self-sampling) versus Group 2. In addition, as women are randomized within each site, sample size calculations were adjusted to account for clustering of patients within each community. Based on these parameters, we examined power estimates under a variety of assumptions for attrition with estimates ranging from 0% to 30%. As shown in Figure 1, with a total sample size of 600 women (200 women in community), we will have sufficient power for the main study hypotheses, under a variety of attrition rate assumptions at a 0.05 level of significance.

#### Participant timeline

At each of the three sites we are recruiting 200 women over a 40-month period with a target of six women per month at each site for a total of 20 women per month. At 6 months following enrollment, we will conduct a follow-up survey to determine if women completed screening for cervical cancer. Participation ends after this 6-month interview.

#### Recruitment

Three CHWs based at the three clinical sites located in our target communities are leading the recruitment effort. With input from local CABs, field supervisors, and University of Miami study team staff, and using techniques such as community mapping, each CHW devises recruitment strategies that are most appropriate to their locale. Examples include targeting high volume areas such as laundromats, pharmacies, hair salons, flea markets, social service sites (WIC centers), libraries, churches, and community colleges. Other strategies include informing the community about the study through radio stations serving ethnic listeners, free community service advertisings in local newspapers, as well as flyers and posters. Once women are identified through these outreach strategies, the CHWs screen them for potential eligibility. We collect de-identified data from all assessments to track the number of women approached by the CHWs and the proportion who were not study eligible due to reasons such as age or having a Pap smear test within the last 3 years. Among those women who have not had a Pap smear, CHWs ask additional questions addressing the inclusion and exclusion criteria. For those women who are potentially eligible, the CHWs describe the study in further detail. If a woman expressed potential interest, we assign a study ID and collect two contact numbers and follow-up with phone calls to arrange a mutually convenient time and location to conduct an intake interview. CHWs make up to 10 attempts to reach each woman. Ideally, the intake visit takes place within 4 weeks of the initial contact. The CHW and RA complete two appointment reminder calls prior to the visit.

### Methods: data collection, assignment of intervention, and analysis

#### Data collection methods: study intake visit

The study intake visit takes place in an area of mutual agreement with the woman. The intake visit most often occurs in the woman’s home or the local health center but may also take place in other locations. During the intake visit, the RA completes another inclusion screening to verify that the woman meets the study criteria. The study is again explained to the woman and any questions answered. After the woman signs the informed consent form, the RA then proceeds with the structured interview. Based on community input, brevity was the overarching principle driving our study intake procedure. For this reason, we limited the intake survey process to take no more than 30 minutes. In Table 1, we list the questions and instruments used in our intake survey. At the conclusion of the intake visit, the RA thanks the woman for her participation in the study and gives her a study business card and a $25 store gift card to cover their time and effort for participation in the study. The RA tells her to expect a call within a week from the CHW to identify her assigned study group. The RA also reminds the woman that at 6 months there will be a follow-up interview.

**Table 1:** **Questions in the SUCCESS intake interview**

Covariates	Questions/Instruments used
Demographics	Age, race/ethnicity, education, income marital status, and citizenship status
Acculturation	Marin-Marin scale [28]
Health literacy	SAHL-S&E [28] (only asked if person are fluent in English or Spanish); not available in Kryeol thus they are only asked if they can read
**Secondary outcomes**	
Access to care (health insurance, having usual source of preventive care, and visit to providers)	Questions from Center for Disease Control National Health Interview Survey Access to Care module
Cervical cancer knowledge, attitudes, and beliefs	Questions from the NCI’s Health Information National Trends Survey (HINTS) [28]

#### Data management

After discussion with some of our community partners and advisory groups, it was felt that collection of data using pen and paper would be more acceptable to the target population than using computer tablets. Thus, we are using paper surveys to collect data which is then input into a Microsoft Access relational study database by a data manager. Initially, all survey entries were double checked for accuracy by a RA and since then every 10^th^ survey is being double checked. Data is also periodically checked by the study statistician for values that are missing, out of range, and for other inconsistencies. As needed, these values are discussed with the study team who then decide how to proceed based on the cause.

#### Allocation and blinding

Once women complete the intake questionnaire they are randomized into one of three possible interventions by the study coordinator who has the pre-generated randomization list from the study statistician. As the study design is hierarchical, with each participant nested within a site, the randomization is based at the site level. A study coordinator notifies the CHW assigned to that woman of the group assignment, and the CHW then follows-up with the women within a week, informing them of their assignment. If women are randomized to Groups 2 or 3, a follow-up visit is scheduled, ideally no later than a month from randomization. The RA who conducts the initial intake visit and 6-month follow-up assessment is blinded to study allocation. However, both women and CHWs are aware of the group to which each participant was randomized.

#### Follow-up evaluation at 6-months

At 6 months, a RA blinded to study group allocation does a 6-month follow-up evaluation. The visit takes place in an area of mutual agreement with the woman. Up to 10 phone calls are made to the patient at different times and days, including weekends, to try to contact them to schedule the appointment. If the participant cannot be reached, attempts will be made using the other contact information that was provided during study intake or follow-up phone calls. If after 2 months the RA has been unable to schedule the appointment, in most cases, participants will be considered as lost to follow-up. However, in some cases the interval may be extended. This may occur if, for example, a participant was out of the country for a few months but had planned to return.

#### Statistical analysis

All data will be analyzed using the intent-to-treat principle and we assume that women who were lost to 6-month follow-up did not have any screening. Initially, we will perform an explanatory data analysis; visually via graphics/plots, such as scatter and box-whisker plots, and numerically by descriptive statistics, such as range, median, means, and standard deviations, for measurements taken on a continuous scale. Percentages and various types of cross-tabulations will be performed for measurements taken on a categorical scale. Corresponding confidence intervals for means and proportions will also be calculated. To have a better understanding of the relationships among different study measurements, parametric or non-parametric correlation coefficients, bivariate, and multidimensional cross-tabulations, scatter, and box-whisker plots will be constructed. Prior to analyses, baseline differences of key covariates from each arm will be examined with respect to our primary outcome, particularly those related to socio-economic status. We will also examine differences on key variables between participants who completed the study and lost-to-follow-up. Analysis of variance for differences in means and *χ*^2^ test for difference in proportions will be used to test the differences among the three study arms. Several univariate and multivariate logistic regression models will be used to calculate odds ratios and corresponding 95% confidence intervals for exploring the result of having had Pap smear screening and the relationship to various types of key study covariates. Two-way interaction terms between various study variables and the study arms will also be included in the models. Standard diagnostic tools will be used to assess model fit. Transformations of the continuous data in order to meet statistical assumptions will be undertaken when indicated. We will treat primary and secondary outcomes as separate clusters, setting a 0.05 level of significance to the primary outcome. A Bonferroni correction will be applied to secondary outcomes. All statistical analyses will be carried out using SAS® version 9.3 or later for Windows (Cary, NC, US) and/or the R project for Statistical Computing for Windows (http://www.r-project.org/).

#### Subgroup analysis

Planned subgroup analyses include examining whether the intervention was more efficacious among women in one community versus another. We will also examine if there was a differential impact of the intervention among women who had lower education, lower acculturation level or had lower levels of baseline cervical cancer knowledge. In addition we will also examine if there was a difference among the groups in awareness of their results and among those told of abnormal findings (Pap or HPV), the proportion having had appropriate follow-up testing. We will consider all these to be hypothesis-generating analysis and not definitive since we will lack statistical power for most of these subgroup analyses.

#### Cost analyses

Although the study does not include a formal cost effectiveness analysis, we will provide estimates of the cost of screening an individual woman in the each of the intervention arms. To calculate a total cost for each group we will assess the resources that were used in the project. This will include all personnel time, including personnel time devoted to training the RAs and CHWs, RA and CHW time (e.g., time spent assessing eligibility, managing contacts, recruiting, conducting the intervention, and administrative time), and resources used such as space, supplies, and medical care. For each of the different types of resource utilization, including personnel time, we will establish a standardized price for a unit of that resource. The cost of the medical care categories will be obtained by either i) the actual cost (e.g., for analyzing the self-samplers) or ii) the average cost of Pap smears at the clinics where testing was done. Total costs for each activity or supply will be calculated by multiplying the units used by the standardized “price”. Costs that are not attributable to a single arm, such as personnel time for training the CHW and RA, will be divided equally among the three arms of the project, or allocated as best estimated by resource use intensity of each arm. Total costs for each of these arms will be calculated using the information on resource utilization and standardized prices. Then an average cost per participant, and per woman screened, for each group can be obtained. From knowing the cost per woman screened in each arm, we can determine the additional cost of screening additional woman in Groups 2 and 3 as compared to the group simply receiving public health outreach.

#### Data monitoring

The NCI expressed high interest in having semi-annual data with respect to our primary outcome as the study was progressing. Thus, interim analyses are being performed every 6 months on our primary outcome and reported back to the NCI. As part of a data safety monitoring plan, at monthly study team meetings attended by all research study staff, the RA and CHWs present any adverse events that resulted from study participation as well complaints by participants and any other unanticipated harms and unintended effects of participating in the study. In addition, our quality control activities include monitoring of recruitment (weekly, using standard reports), monitoring the eligibility of enrolled participants, and periodic audit of consent forms to insure signatures and properly filed consents for all enrolled participants.

### Ethics and dissemination

#### Protocol approvals and amendments

The study was approved by the University of Miami Institutional Review Board, initial approval date on 12/22/2010, protocol #20100834. Since then, there have been eight approvals for minor amendments to the study protocol, most of which involve adding study personnel to the research team.

#### Consent

Informed consent is being obtained by our two bilingual RAs (Spanish/English, Haitian Kreyol/English) prior to the baseline intake. The women are given the informed consent form in their preferred language which they read and review along with the RA. If the woman states that she cannot read or if the CHWs suspects that the woman may not be able to read then all of the information in the consent form is read aloud to them. Once all questions have been answered, the woman is asked to sign the informed consent form. For women who cannot read, a witness signs indicating the form was read to the participant.

#### Confidentiality

To maintain participant confidentiality, study specific ID codes are used in all study tracking and analyses. Only the CHWs and project coordinator have access to the files linking participants with study ID numbers. These password protected files are stored in password protected computers. All paper surveys only have study ID numbers without names. These are stored in locked cabinets in locked offices.

#### Financial interests

None of the study team members, including the principal investigators, have any financial or other competing interests in the study.

#### Access to data

Only the study statistician has access to the raw data files entered by the data manager. Data files that have been cleaned and appropriately grouped and categorized will be made available to study personnel requesting such data. This includes the principal investigators, project coordinators, and our community partners. The statisticians will also make preliminary charts and tables when requested by study team members. Outside groups interested in access to the data will be granted access to limited datasets after submitting such requests to the study principal investigators.

#### Post-trial care

Ensuring that at the conclusion of the study all unscreened women are offered recommended screening was an important ethical consideration made by our community partners. Thus, once the study-related follow-up data has been collected, the CHWs will offer HPV testing to all women who have not been screened. For unscreened women declining the HPV testing, the CHWs will also offer their assistance in making appointments for Pap smears. For tracking purposes, we will also collect data on HPV outcomes among these women.

#### Dissemination policy

Our study findings will be presented at scientific meetings and published in peer reviewed journals. Based on our community-based participatory research framework, the active role of the community partners is such that we have agreed to have at least two partners play a key role in drafting each manuscript that will be published from this research effort and appropriately include them as authors in all such manuscripts. The NCI will also receive copies of our final results in our final report. Findings will also be reported in http://clinicaltrials.gov/. In addition, we will also present our results to community advisory boards we established in each community. We will also disseminate our findings to community members, patients, and other stakeholders, as well as in public forums, presentations to their boards, and lay summaries in the newsletters and ethnic media such as radio and television in all three languages (Spanish, Kryol, and English).

## Discussion

Minority immigrant women (both Hispanics and Blacks from Haiti) are less likely to be screened for cervical cancer than the general population. In this research project of the South Florida Center for the Reduction of Cancer Health Disparities (SUCCESS) we are conducting a randomized study on cervical cancer screening among 600 women in three underserved communities in Miami-Dade County. Data from this study will allow us to determine optimal approaches to increase cervical cancer screening among underserved women in our community. It will compare screening through standard public health outreach versus CHW-assisted patient navigation and a CHW intervention that includes the option for HPV self-sampling.

One methodological concern relates to our study design in which we have assigned one CHW to each community. As a result, the differences observed across different communities may reflect the impact of the CHW and not the intervention itself. Process data such phone calls, time with each participant, and follow-up will allow us to examine service intensity and examine if the intensity of the intervention varied in each community. In addition, as both groups two and three involve delivery of similar educational content, as a fidelity check of the CHW intervention we can examine differential changes in cervical cancer knowledge across the three sites. If present, such variations, may suggest differences in the CHW delivery of the intervention.

A second important methodological concern is that our primary outcome is a self-reported Pap smear, which is the approach used to track cervical cancer screening in national surveys such as the Behavioral Risk Factor Surveillance System and National Health Interview Survey. However, self-reports are a subjective measure and prior studies suggest that when initially asked about cervical cancer screening using self-reports, women may overestimate having a Pap smear by nearly 30 percentage points [29, 30]. However, those studies focused on women initially being asked and not those being assessed on a follow-up evaluation. For our study, we prioritized consistency in how our primary outcome was assessed initially and at 6-month follow-up. Further, in our study, women had many options where to obtain a Pap smear. These included FQHCs, public hospitals clinics, free clinics, Center for Disease Control-funded programs, and private providers. Obtaining access and querying the electronic medical records or manual chart review in each of these providers systems would have been administratively challenging. For these reason, we opted self-report as our primary outcome.

An additional concern relates to the evidence in support of HPV screening. Although HPV detection is gaining increased acceptance as a primary screening strategy, self-sampling has still not received full endorsement from the USPSTF. Instead, self-sampling is recommended as an option to be considered in hard-to-reach populations [28]. Having underserved women be screened through an intervention that has not been fully endorsed by the USPSTF raises issues of social justice and access to care inequities. The Affordable Care Act is an important step in helping improve access to care among many women. However, some low-income women residing in states that have opted not to expand Medicaid, such as Florida, and women who are undocumented will not benefit from the Affordable Care Act insurance expansions. Until all women have equitable access to care, the approach we propose is preferable to not having women screened. Additionally, our group continues to explore other approaches to expand cervical cancer screening. For example, we recently obtained an award from the National Cancer Institute to examine if HPV self-sampling kits distributed by CHWs at health fairs and mailed back are an effective screening option. Contributing towards the science of ensuring all women receive adequate cancer screening is an important contribution our SUCCESS center is making towards reducing and ultimately eliminating cancer inequities.

## Trial status

As of May 9, 2014, we have enrolled 582 of our target 600 women into the study.

## Abbreviations

CAB: Community advisory board
CHW: Community health workers
FQHC: Federally qualified health center
HCN: Health Choice Network of Florida
HPV: Human papilloma virus
NCI: National Cancer Institute
PCR: Polymerase chain reaction
RA: Research assistants
SUCCESS: South Florida Center for the Reduction of Cancer Health Disparities
US: United States
USPSTF: Unites States Preventive Services Task Force.
